# How Mitochondrial Signaling Games May Shape and Stabilize the Nuclear-Mitochondrial Symbiosis

**DOI:** 10.3390/biology13030187

**Published:** 2024-03-15

**Authors:** Will Casey, Thiviya Kumaran, Steven E. Massey, Bud Mishra

**Affiliations:** 1 Cyber Science Department, United States Naval Academy, Annapolis, MD 21402, USA; austincasey@gmail.com; 2 Courant Institute of Mathematical Sciences, New York University, New York, NY 10012, USA; tk3101@nyu.edu (T.K.); mishra@nyu.edu (B.M.); 3 Biology Department, University of Puerto Rico—Rio Piedras, San Juan, PR 00931, USA

**Keywords:** signaling game, mitochondria, endosymbiosis, information asymmetry

## Abstract

**Simple Summary:**

We introduce a biomolecular signaling games model of cell-mitochondrion interaction, that helps to explain how cooperation between the two may have become established and maintained.

**Abstract:**

The eukaryotic lineage has enjoyed a long-term “stable” mutualism between nucleus and mitochondrion, since mitochondrial endosymbiosis began about 2 billion years ago. This mostly cooperative interaction has provided the basis for eukaryotic expansion and diversification, which has profoundly altered the forms of life on Earth. While we ignore the exact biochemical details of how the alpha-proteobacterial ancestor of mitochondria entered into endosymbiosis with a proto-eukaryote, in more general terms, we present a signaling games perspective of how the cooperative relationship became established, and has been maintained. While games are used to understand organismal evolution, information-asymmetric games at the molecular level promise novel insights into endosymbiosis. Using a previously devised biomolecular signaling games approach, we model a sender–receiver information asymmetric game, in which the informed mitochondrial sender signals and the uninformed nuclear receiver may take actions (involving for example apoptosis, senescence, regeneration and autophagy/mitophagy). The simulation shows that cellularization is a stabilizing mechanism for Pareto efficient sender/receiver strategic interaction. In stark contrast, the extracellular environment struggles to maintain efficient outcomes, as senders are indifferent to the effects of their signals upon the receiver. Our hypothesis has translational implications, such as in cellular therapy, as mitochondrial medicine matures. It also inspires speculative conjectures about how an analogous human–AI endosymbiosis may be engineered.

## 1. Introduction

The endosymbiosis of the alpha-proteobacterial ancestor of mitochondria into a proto-eukaryote represents a key step of eukaryogenesis [1]. This event appears to have led to an adaptive radiation producing the wide diversity of extant eukaryotic lineages, as alternative amitochondrial lineages leading from the proto-eukaryote are absent. Consequently, the nuclear–mitochondrial symbiosis laid an underlying protocol, upon which staggering eukaryotic diversity has been built, including subsequent or quasi-concurrent endosymbioses, including chloroplasts. While mitochondria contribute to the cell metabolically via oxidative phosphorylation, they also have an important role in apoptosis and aging in animals. Given the fundamental nature of the “protocol”, it is unsurprising that the nuclear–mitochondrial relationship has a key, if often underappreciated, role in the etiology of numerous human diseases [2]. In particular, mitochondrial informational integrity is a frequent factor in disease development [3,4,5,6].

Thus, we believe that to classes of data used in whole-genome association studies for population genetics and disease studies, one must add information about mitochondrial heteroplasmic genomics and their effectiveness in interpreting phenomena such as apoptosis, stem cell replacements, host genome polymorphisms, mutations and error repair, senescence, autophagy (mitophagy) and mitochondrial fission–fusion processes. Better understanding these dynamics will help us address diseases such as cancer, neurofibromatosis type 1 (NF1), rare Mendelian disorders, neurodegeneration (Parkinson’s, Huntington’s and Alzheimer’s Diseases, and amyotrophic lateral sclerosis (ALS)), and possible cellular therapies to be built upon chimeric antigen receptor T-cell (CAR-T) [7], stem cells [8], and clustered regularly intersperse short palindromic repeats (CRISPR)-based gene editing [9]. 

However, given its antiquity, and the lack of intermediates and alternative lineages (representing in some senses a “singularity”, “frozen accident”, “Cambrian-like explosion”, “pop hypothesis” or “big bang”), the onset of the mitochondrial endosymbiosis still remains poorly understood. Connected with this sudden emergence, the reason the symbiosis has remained stable for so long (up to 2 billion years [10]) is of interest. While Cosmides and Tooby posited that the mitochondrial–nuclear interaction is not always perfectly aligned, with the possibility of mitonuclear genomic conflict [11], this is likely mostly limited to the effects of maternal inheritance on sex ratios.

Previously, we developed and explored a sender–receiver signaling games model of biomolecular interactions [12,13,14]. Signaling games provide a novel means of understanding cooperative and parasitic molecular interactions, as they do for cooperative and parasitic interactions at the organismal scale [13,14]. Cooperation is based on trust, which may be established via the observation of signals sent by a sender to a receiver. Hence, signaling games may provide insights into nuclear–mitochondrial symbiosis, which is mainly cooperative. Here, we use signaling game simulations to investigate the impacts of cellularization on the relationships between prokaryotes and a proto-eukaryotes, with a focus on the development of trust signals (e.g., signaling leading to correlations of encounter) between the two organisms. Speculatively, we hope that this model will generate novel engineering approaches to induce human–AI symbiosis with well-designed, more affordable signaling (e.g., tying hands, sunk-, installment- and reducible-costs among nation states) [15]. 

## 2. Methods

Previously, we described a framework for biomolecular signaling games, which involved a sender gene that expresses a biomolecular (protein or RNA) signal that then interacts with a biomolecular receiver (coded for by a corresponding receiver gene) [12,13,14]. Upon receipt of the signal, the receiver biomolecule undertakes an action that produces overall fitness (utility). In a cooperative interaction, the biomolecular signal is honest and fitness accrues to both sender and receiver genes. However, in a parasitic interaction, the biomolecular signal is deceptive, and results in fitness accruing to the sender gene, at the expense of the receiver gene. Such a deceptive biomolecular signal often involves molecular mimicry [14].

**Figure 1:**
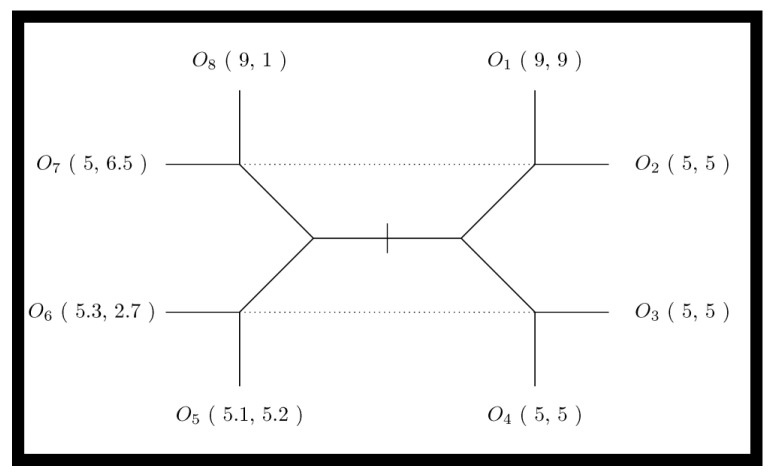
The signaling game extensive-form game tree. The extensive-form game tree describes the outcomes of the signaling game as *O*_1_ through *O*_8_. Each outcome confers differential rewards to sender *S* and receiver *R* agents as (*U_S_*, *U_R_*). The sender *S* will have a type (determined by nature); honest types result in outcomes to the right of the center (i.e., outcomes *O*_1_ through *O*_4_), while non-honest types result in outcomes to the left of center (i.e., outcomes *O*_5_ through *O*_8_). In signaling games, the receiver will remain unaware of the sender’s true type but will perform an action when they receive the sender’s signal. Senders will signal one of two messages; in the extensive-form game tree the message variant one is indicated by all outcomes above the midpoint, (i.e., outcomes *O*_1_, *O*_2_, *O*_7_, and *O*_8_), while message variant two is indicated by all outcomes below the midpoint (i.e., outcomes *O*_3_, *O*_4_, *O*_5_ and *O*_6_). Next, the receiver selects one of two differing actions; action one is indicated by vertical leaves (i.e., outcomes *O*_1_, *O*_4_, *O*_5_, and *O*_8_), while action two is indicated by horizontal leaves (i.e., outcomes *O*_2_, *O*_3_, *O*_6_ and *O*_7_). Finally, game outcomes for sender–receiver pairs depend on a combination of sender type, sender message, and receiver action. Dotted lines indicate the informational constraints of the receiver. Since the sender is either honest or otherwise, but the receiver can only distinguish the sender’s signal, the dotted lines indicate the information available to the receiver when they act. The left and right halves of the extensive-form game tree thereby represent the two “possible worlds” the receiver is in when they must select their action.

**Figure 2:**
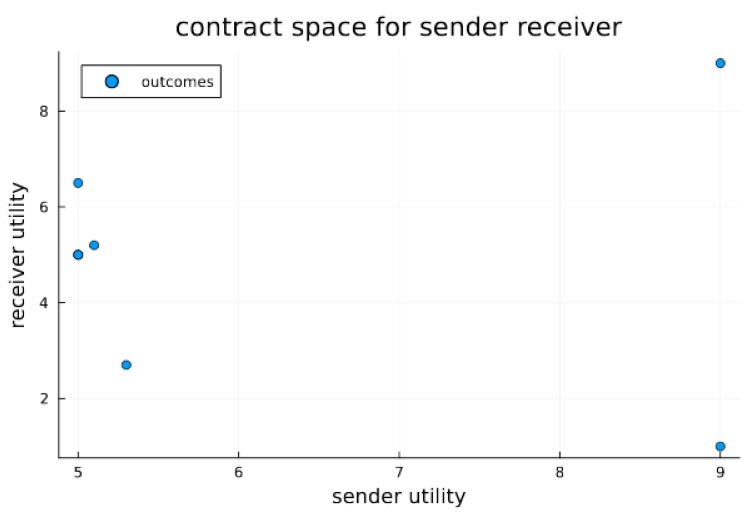
Contract space for signaling game. Contract space illustrates the joint utility outcomes for signaling games for both sender and receiver. The Pareto optimal outcome *O*_1_ is clearly visible as the best and most preferable outcome for both the sender and receiver; however, without a stabilizing or mediation mechanism, it is not necessarily in equilibrium. In the experiments below, we test whether endosymbiosis, which results in strongly codetermined outcomes, can furnish stability (i.e., equilibrium) for O_1_. We do this by comparing to a system of extracellular agents and observe the differing propensities for outcomes.

The essential interaction is thus a signaling game (see Figure 1). The Endosymbiosis Signaling Game then considers a possible cellularization constraint for sender/receiver pairs; this constraint induces repeated interactions between the sender/receiver endo-pair and imposes a common or inclusive utility. Additionally, endosymbiosis will introduce a novel population of cellularized pairs. The utility parameters for signaling games are also visualized in contract space for the sender and receiver (see Figure 2). Contract space provides the clearest view of the Pareto Efficient outcome O_1_; however, the question remains—what, if anything, can stabilize the Pareto Efficient outcome when sender signaling strategies can dabble with deception in order to obtain similar satisfaction at the expense of the receiver? To examine how cellularization can modulate the dynamics of signaling games, we have designed a mathematical simulation expressing the sender and receiver agents found in various states of endosymbiosis. The varied states of communicating agents (i.e., sender and receiver) range from agent independency relying on single-shot games among randomly encountered agents, to a state of complete codetermined encounters (a state inhabited by sender and receiver agents within a common cell). *Agent Types* include sender (*S*), receiver (*R*), and endo-pair (*E*), composed of one sender and one receiver. The sender can be considered a proto-mitochondrion, while the receiver is a proto-eukaryote. Our simulation models a population of the three agent types, with transitional probabilities of *α* that an independent sender/receiver pair transitions to an endo-pair, and *β* that an endo-pair will transition into an independent sender and independent receiver. *Agent Utility* for sender–receiver agents is provided in the extensive-form game tree shown in Figure 1. Note that within endo-pairs, sender and receiver utilities are aggregated to form a completely inclusive utility for the cell or the cellularized combination of the sender and receiver within the endo-pairing. *Domain* for the model includes a spatial domain *D* with active populations of all three agent types in each location, indexed spatially in two dimensions by *d*(*i*,*j*). While the population summed over *D* will remain fixed (under the fixed population size assumption), variations in location populations are possible with a dispersion parameter that acts on each agent as a Bernolli trial with parameter *γ*, along with a random walk type function that determines the next position based on the current position. *Encounters* occur between sender and receiver types and depend on agent cellularity status. Independent sender and receiver agents are randomly paired into encounters for each position of the domain. For endo-pairs, the sender and receiver remain in protracted encounters throughout the life of the co-inhabited cell. Each encounter results in a signaling game play. For independent senders and receivers, the number of encounters is modeled as equal to the smaller of the two populations. *Rewards* are determined for each agent within each signaling game. Note that for endo-pairs the reward to the inhabited cell is the joint aggregate reward. *Strategy*: Senders have two natural types, each of which have two signaling strategies. Receivers can act in one of two ways, which depends on the sender’s message, thus four receiver strategies are available. The cellularized endo-pairs, having both a signaling and receiver agent, fit into one of eight possible strategic types. *Replication* depends on signaling game rewards within each population—independent sender, independent receiver and endo-type. At each generation, for each location, for each population, rewards are aggregated by strategic type. This formulation implies that for the population of independent senders, the rewards earned via each strategy type are aggregated, thus each of four independent sender strategic types receives a score (at each location for each generation). Similarly, rewards are aggregated for each of the four independent receivers’ strategic types, and for each of the eight endo-pair strategic types. Once rewards are aggregated, the reward normalized probability vector is used to resample the population as a type of replication. We let *U_ijk_*(*s_h_*) be the aggregate reward of sender strategic type (indexed by *h*) in the domain position (*i*,*j*) at generation *k*, and *U_ijk_*(*R_g_*) likewise be the reward of the receiver strategic type (indexed by *g*) in the domain position (*i*,*j*) at generation *k*, while *U_ijk_*(*E_f_*) is the reward of the receiver strategic type (indexed by *f*) in domain position (*i*,*j*) at generation *k*. Letting *n_ijk_*(*S_h_*), *n_ijk_*(*R_g_*), and *n_ijk_*(*E_f_*) be the counts of various strategic types of sender, receiver and endo agents at location (*i*,*j*) at time *k*, the populations then composed of numbers of senders, receivers, and endo types at the same coordinates are given by: *n_ijk_*(*S*) *= Σ_h_n_ijk_*(*S_h_*), *n_ijk_*(*R*) *= Σ_g_n_ijk_*(*R_g_*), and *n_ijk_*(*E*) *= Σ_h_n_ijk_*(*E_f_*). The replication process reproduces new populations at time *k +* 1 from populations at time *k*. For each population *X ∈ {S*,*R*,*E}*, we recreated each population for generation *k +* 1 by sampling the Dirichlet multinomial:

$$\begin{array}{ll} P(n_{ij(k+1)}\left(X_{v}\right) & =x\;;{\left[U_{ijk}\left(X_{v}\right)\right]}_{v})\\ & =\frac{\Gamma (\sum_{v}\;n_{ijk}(X_{v})+1)\;\Gamma \left(\sum_{v}U_{ijk}\left(X_{v}\right)\right)}{\Gamma \left(\sum_{v}\;n_{ijk}\left(X_{v}\right)+\sum_{v}U_{ijk}\left(X_{v}\right)\right)}\;\prod_{v}\left(\frac{\Gamma \left(n_{ijk}\left(X_{v}\right)+U_{ijk}\left(X_{v}\right)\right)}{\Gamma \left(\;x+1\right)\Gamma \left(\;U_{ijk}\left(X_{v}\right)\right)}\right) \end{array}$$

Replication can most simply be understood as a multiplicative boosting/attenuation process (replicator dynamics), where, for population *X*, the probability that a replicant is type *v* will be: *U_ijk_* (*X_v_*)*/*(*Σ_v_*(*U_ijk_* (*X_v_*))). Said differently, strategic types that perform better than average are likely to be amplified, and ones that do worse than average are likely to be attenuated; this is because the expected replication for strategic types is proportional to the rewards they gained in the prior time step. The *mutation* of agents occurs after replication; for each agent a Bernoulli trial with parameter *μ* will determine if it is reconstructed by uniform random sampling over the possible strategy types available to each type. The *dispersion* of agents occurs after mutation; for each agent a Bernoulli trial with parameter *γ* will determine a random move to a neighboring domain location. At the completion of this step, the time index is advanced and we continue by repeating the encounter step.

### Limitations

Our model makes several simplifying assumptions to enable our computational approach. These assumptions may limit the model’s realism. In our model, the cellularization and decellularization processes are acting in equilibrium to maintain fixed population size. While the fixed population size assumption is frequently utilized for population simulation studies, the back and forth configurational changes for sender/receiver pairs transitioning from independent agents into endo-paired cell agency are more novel, presenting some limitations.

First, cellularization is widely considered a one way transition, and our model may unrealistically assume that decellularization occurs too frequently. We designed our model to test the essential game of reconfigurations for sender/receiver agents with respect to cellularity. Such testing of alternate configurations for agent utility (i.e., independent vs. cellular condition) can be done so long as a portion of the population conducts cellularization experiments while a portion of the population refrains from cellularization. To maintain reasonably constant portions of the population in various conditions and under a fixed population size hypothesis for sender and receiver agents, we introduce the decellularization process. While our decellularization process could be given a much lower rate parameter, the simulation would then require a far greater number of agents and a longer runtime such that that the population can reach stable portions in and out of the cellular condition. As such, our unrealistically high rate of decellularization is intended to expedite a fixed population reaching stable portions of agents under various cellular conditions. While this is a limitation, simulations that accurately model the raw numbers of independent sender/receiver agents required for more realistic parameters also face computational limitations. We select this tradeoff as the best option to examine the essential question of cellular configuration vs. non cellular configuration.

Secondly, the question remains that, if cells can capture a single sender, why not multiple senders? Our model is designed to consider the simplest case of one receiver paired with one sender in the cellular form. By addressing this base case, we can further enable enhancements that potentially scale in this way by considering configurations of multiple senders with one (or possibly multiple) receiver(s). We plan to address these more complex cases in future work, and here only offer the base case.

## 3. Results

Following the methodology outlined above, an insightful set of parameters for the replicator dynamic system are summarized in Table 1.

In addition to the parameters above, additional parameters are entered through two orthogonal simulation components—first, the signaling game parameters that are specified in Figure 1; second, the encounter process whereby we follow a simple procedure of pairing sender/receiver pairs for signaling games, as described in the Methods. Note that the simulation is initialized without endo-pairs, i.e., all initial agents are independent sender/receivers. For a discussion of how rates *α* and *β* yield a queuing process, resulting in a stationary distribution of the three agent types, see the Supplementary Materials. The initial population is assembled by repeating a uniform random selection of a sender or receiver agent type, followed by a uniform random selection of strategy type given the agent type, and then a uniform random selection of domain location.

Statistically, the expected split in the population is an equal division of sender and receiver agent types. As mentioned, the proportion of endo-pairs will stabilize (see Supplementary Materials) after a burn-in period. In our experiment, we keep it simple by using a single domain location, i.e., a domain with one location, thereby nullifying the action of dispersion as agents selected for dispersion with rate *γ* will be confined to the singular location. Adding the dispersion process results in highly interesting temporal–spatial dynamics; however, these are unnecessary for the current demonstration.

**Figure 3:**
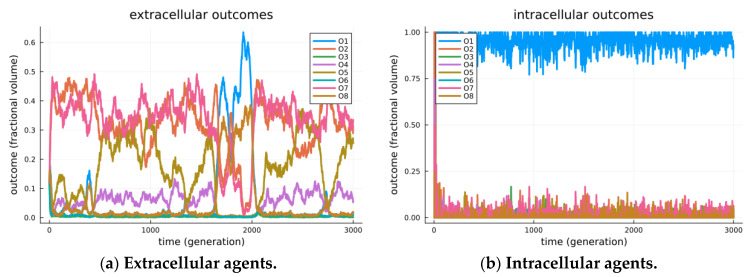
Observed outcomes for simulated signaling games. Simulated outcomes for a simulation of 10,000 agents over 3000 generations. Agents are initially created by randomly selecting either a sender or receiver type. Initially, all agents are extracellular and randomly encounter other agents. Each encounter of extracellular agents will include a Bernoulli trial that the sender and receiver will cellularize (*α* = 0.0001). Senders and receivers in a cellularized state will no longer encounter random agents, but rather encounter each other repetitively. Further senders and receivers in a cellularized state can decellularize with a Bernoulli trial (*β* = 0.01), thus returning the sender and receiver agents to the extracellular state. Note that the two processes will reach a stationary distribution, maintaining a nearly fixed proportion of all sender/receiver agents within the inter cellular arrangement. In (**a**), we observe the system restricted to senders/receivers in the extracellular state. Extracellular sender and receiver agents exhibit a variety of modes; however, stability for O_1_ is inherently limited and mitigated by the strategic deception of senders. In the extracellular world, senders and receivers are separate populations, each of which undergoes replication dynamics that are relative to others in their population. A variety of outcomes including *O*_1_, *O*_2_, *O*_7_ and *O*_8_ were found to be most frequent in an episodic manner. In (**b**), we observe the system restricted to senders/receivers in the intracellular state. The intracellular state represents a separate population, where the replication of senders/receivers undergoes replication dynamics that depend on joint utility. As such, Outcome *O*_1_, found to be the most frequent outcome, appears evolutionarily stable, and this suggests that endo-symbiosis is achieved by stabilizing the Pareto efficient outcomes.

The results are visible in Figure 3, where separate visualizations are shown for the agents in extracellular states (a) and those in cellularized states (b). In the extracellular state, the system displays a variety of modes, as illustrated by the episodic maximally expressed game outcome (among all encounters at a given time). We see in Figure 3a one period where *O*_1_ is the mode (generations ~1700 through 2000); however, its stability cannot be maintained, as when a critical threshold of *O*_8_ outcomes arise, the best receiver strategy crosses over to a less beneficial checking strategy, *O*_2_. Still, the modes are seen to transition over time, including episodes where outcomes *O*_1_, *O*_2_, *O*_7_ and *O*_8_ are the maximally expressed game outcomes (modes). These transitional and transient effects are detailed as *adversarial chase* in our earlier studies investigating the roles of signaling games in biological organization [14].

In Figure 3b, we observe the strong modality of cooperation, i.e., the Pareto efficient *O*_1_ outcome, as the maximally expressed game outcome. This is even seen to occur before the endo-pairing population reaches equilibrium. We reason that as soon as endo-pairs arise, any combined strategy that achieves the utility of *O*_1_ is so much more beneficial than other outcomes that it is immediately amplified (by similar proportional rates) in the replication process. Thus, the replication of similar strategies (that achieve outcome *O*_1_) will immediately take over, leaving only two sources of variability: first, the uptake of endo-pairs from the extracellular environment, and second, the mutation process, which randomly re-selects endo-pair strategies. Combined sources of variability (at presented parameters) are incapable of displacing the evolutionarily stable strategy of *O*_1_ for endo-pairs.

### Signaling Game Equilibria

**Figure 4:**
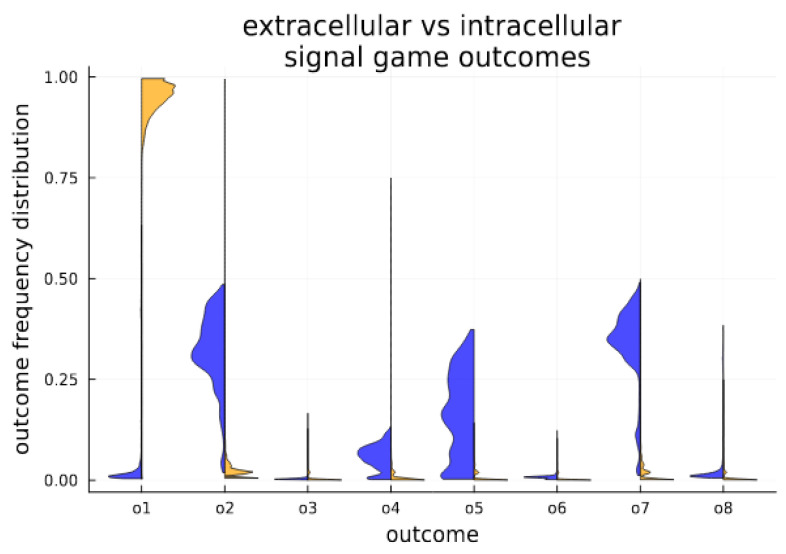
Cellularization enables stability for Pareto efficient outcomes. Outcome expression profiles are compared for extracellular (blue) and intracellular (orange) conditions in the simulated system. Above each game outcome *O*_1_–*O*_8_, we plot a histogram showing the distribution of outcome expression as a fractional volume of all outcomes. The histograms aggregate measures (volumetric fraction of outcomes) over time (number of generations out of 3000 generations). They provide a visualization of time in state, and clearly indicate that the cellular state strongly promotes the Pareto efficient outcome *O*_1_, which is far less frequent among senders and receivers in the extracellular populations.

The dynamic nature of signaling is divided by cellularization state. In the intracellular environment, a separating equilibrium is obtained, characterized by the strong stability of an efficient outcome. In the extracellular environment, various forms of separating, partial pooling and babbling are witnessed; see Figure 4 for the time average in each state.

## 4. Discussion

Within our simulation experiment, cellularization appears to provide a protective barrier for dynamic equilibrium. Within cellular environments, a strong form of separating equilibrium for signaling games is established, while outside the cellular environment a less stable environment is observed to display a variety of states and equilibria transitions. In evolutionary signaling games, separating equilibria are important for the implementation of cooperative interactions between replicating individuals.

The simulations show that under reasonable parameter choices, the cell wall establishes a barrier enabling a new equilibrium within cells (separating equilibria), while babbling (or at best, episodic separating/pooling/babbling, as described in [14]) is the norm exterior to cells. We reason that the strong separating equilibrium arising in the cellular environment is a product of the sample-and-hold effect of repeated interactions, the inclusive utility or common destiny constraints of the sender/receiver, and replication in the novel cellular lineage. As such, the outcome modality arising from these stabilizing mechanisms could enable endosymbiotic exploration that is not possible in non-cellular environments when the stability of a Pareto efficient outcome is sustained for both the sender and receiver. The establishment of trust between players, and the resultant development of mutualistic cooperation, is facilitated by proximity and repeated interactions, which allows the observation of counter-party behavior and the consequent updating of priors.

Is there something special about mitochondria as mutualistic endosymbionts? Their characteristic features are that they display uniparental (e.g., matrilineal) inheritance; they avoid heteroplasmy [16]; they employ a fission–fusion process, maintaining a controlled range of copy numbers [17]; they signal by metabolic and synaptic spiking [18]; and they use quantum tunneling of electrons to precisely drive ATP pumps in a respiratory chain [19]; But why did they develop these singularly obsessive signaling modalities? What are the full evolutionary roles of apoptosis, senescence, stem cell regeneration, mitophagy and stringent homoplasmy, which go hand-in-hand with the evolution of multicellularity?

Thus, while strategic noncooperative games (with replicator dynamics) have been used to understand evolution, information-asymmetric games at the molecular level promise clearer novel insights into endosymbiosis. This refocuses our attention on the multifaceted translational implications.

1. Aging—The adversarial chase dynamics involving mitophagy, senescence, stem cells, apoptosis, necrosis and heteroplasmy reveal their effects via aging in multicellular organisms. Even if apoptosis and stem-cell replacement could in principle promise unbounded longevity, in reality, stem cells in the niche could act as deceptive mutants unwilling to participate in costly signaling (cowardly Casanova conjecture (Private communication, M.Wigler)) and lead to cancer and death. Telomere shortening and double-stranded breaks (in the lagging strand) could lead to complex genome repair, but there remains a hard-limit to lifespan that could not escape the constraints imposed by mitochondrial surveillance. In tissues where apoptosis is not feasible (e.g., neurons, muscles and heart), mitophagy may be necessary, leading to other manifestations of aging;
2. Genetics and genomics—To understand the aging phenotypes and their connections to the genotypes, Crick’s Central Dogma and the information flow model postulated by it will need to be further extended. This may require novel algorithms and analyses involving copy numbers of mitochondria. So far, mitochondria have been somewhat overlooked. The modern synthesis (uniting Darwin and Mendel) and Crick’s dogma address the context set by the conventions and constraints agreed between mitochondrion and nucleus. While nuclear genomics data have been growing exponentially and ubiquitously, there is a dearth of data available to model heteroplasmic mitochondrial genomics;
3. Technologies—Thus, for a proper translational impact, we would need to perform noninvasive measurements of mitochondrial heteroplasmy by developing systems for (long or medium-range reads) sequencing and mapping. Atomic force microscopy (AFM)-based nanomapping [20] with the imputation of cells of origin of heteroplasmy could offer the breakthrough required;
4. Therapies—As we approach new therapies involving modified nuclear genomes, which generally go by the name “cellular therapies”, we will need to pay special attention to the cells’ mitochondria. These therapies will have a significant impact on IVF (*in vitro* fertilization), stem cell therapies, CRISPR editing and CAR T-based immuno-therapies;
5. Diseases—The approaches suggested would change our understanding of a whole range of diseases, such as NF1 (a rare disease involving young adults [21]), neurodegeneration (Parkinson’s, Huntington’s and Alzheimer’s Diseases, and ALS), and cancer. An interesting application could involve a combination therapy synergizing with DDW (Deuterium Depleted Water,) which could affect mitochondrial respiratory chains [22].

The game theory developed here is still preliminary and nascent; the next natural step in modeling eukaryotes under this framework would be the formulation of a mean field game. Given the enormous number of cells involved in the system, repeated games with discrete agents would be better characterized by each agent simply taking into consideration the mean behavior of all agents in the system. Consequently, the solution to such a game is a strategy that depends only on environmental conditions and the agent’s own choice function.

As hinted earlier, one of the most promising avenues of research resulting from this paper is the relationship between symbiosis and aging. An optimal control approach to explaining aging suggests that there exists a threshold at which it is optimal for the body to allow cell death instead of repairing damaged cells [23]. This threshold is in part determined by a repair cost dependent on the senescence of cells, which we now postulate to be the result of a signaling game. It would therefore be pertinent, when studying processes such as aging and autophagy (and possible remedies), to consider how changes to the mitochondria and its optimal solution in a mean field game affect the repair and death of cells in the system. The relationship between aging processes and mass effects of equilibria (or loss thereof) is an interesting topic for future work, and an area of further development of the game theory model. Cellular senescence and mitochondrial dysfunction (i.e., overproduction of reactive oxygen species) can be viewed as receiver and sender departures from the ancestral signaling equilibrium, and diminish the advantage of cellularity (cheap or cowardly Casanova). The game theory model offers a practical means to interpret the various effects that impair cellular functions, as well as the counteracting processes preventing impairment.

Another (highly speculative) extension to this framework would be the consideration of quantum signaling games [24], and their role in the possible entanglement of mitochondria in a single cell, as well as quantum error correction via mitophagy. But it is entirely possible that traditional classical mechanics already holds the answer, and speculative hypotheses could be “not even wrong”, thus not falsifiable.

Our information asymmetric model provides a new avenue for game theory, which can be applied to generative AI. Mitochondrial endosymbiosis inspires a potential analogy with AI (uninformed receiver) and (informed sender) humans; the extensive-form game we propose will only need minor modifications to suggest how AI–human symbiosis could be engineered to reach well-aligned separating equilibria. The game theory model could provide bio-inspiration for engineering and design problems, whereby Artificial Intelligence offers both risk and reward.

Finally, it is worthwhile to remind the readers about the computational evolutionary work of Nils Barricelli on cellular symbiogenesis on ENIAC, one of the first general-purpose computers at Princeton [25]. With this research, Barricelli opened up the field of computational biology, with the emergence of Artificial Life (AL), Self-reproducing Automata (John von Neumann) and molecular biology (Sydney Brenner and Francis Crick). We extend and differ from Barricelli’s work by connecting it directly to the game theory of von Neumann, while developing it within the context of information asymmetry.

## 5. Conclusions

Here we have described a biomolecular signaling games model of host-mitochondrion interactions. Using simulation, we establish that cellularization promotes the establishment of a separating equilibrium between host and mitochondrion, which is important for the initiation and continuation of the symbiosis. We posit that a number of diseases with a mitochondrial component may involve disruption of the separating equilibrium.

## Figures and Tables

**Table 1:** Parameters for the signaling game simulation.

Parameter	Value	Significance
Total agents	10,000	Fixed population size
Generations	3000	Time duration of the simulation
Signaling Game		Component specified in Figure 1
Encounters		Component specified in methodology
Domain size	(1, 1)	Singular domain (a single location) for all interactions
Cellularization rate: *α*	0.0001	Bernoulli trial for transition to endo-pair, applied to encounters
Decellularization rate: *β*	0.01	Bernoulli trial for transition to independent sender/receiver, applied to encounters
Mutation rate: *μ*	0.01	Bernoulli trial for uniform random reselection of strategic type
Dispersion rate: *γ*	0.01	Dispersion rate, for the Bernoulli trial triggering a random movement to adjacent domain location

## Data Availability

Code can be found at: https://github.com/austincasey/mito_signaling_game/tree/main (accessed on 11 March 2024). Additional in formation can be found in the Supplementary Materials.

## Supplementary Materials

The following supporting information can be downloaded at: https://www.mdpi.com/article/10.3390/biology13030187/s1, Supplementary Material: Source code.
