# Supplementary material for: How Mitochondrial Signaling Games May Shape and Stabilize the Nuclear-Mitochondrial Symbiosis
Source: Biology (Basel). 2024 Mar 15;13(3):187. doi: 10.3390/biology13030187 (PMC10968254; doi:10.3390/biology13030187)
Supplement: Supplementary file 1 [file biology-13-00187-s001.zip › biology-2874291-supplementary.pdf]

Supplementary Material
Source code is available and shared as open source.

Simulation experiments are designed in the Julia programming language. Interactive components of the code can be explored using the pluto notebook addition to the Julia programming language.

We have placed the code at the following location:

https://github.com/austincasey/mito_signaling_game/tree/main

Stationary distribution of cellularization/decellularization queuing process.
These processes play out over the encountering pairs, with probability rate α encounters of independent sender/receiver pairs cellularize to form an endo-pair, and with probability β *an endo-pair decellularized into independent agents*. Letting $f_1(k)$ the fraction of extracellular encounters, and $f_2(k)$ be the fraction of cellular encounters (endo-pairs) at time step k, the transition process is described by the column stochastic matrix:

$$\begin{bmatrix} f_1(k+1) \\ f_2(k+1) \end{bmatrix} = \begin{bmatrix} 1-\alpha & \beta \\ \alpha & 1-\beta \end{bmatrix} \begin{bmatrix} f_1(k) \\ f_2(k) \end{bmatrix}$$

The Perron–Frobenius theorem applied to positive matrices asserts the existence of positive leading eigenvalue, within the theory of markov chains the leading eigenvalue of column stochastic matrices is known to be one. The eigenvalue decomposition of the stochastic matrix can be shown to be:

$$\begin{bmatrix} 1-\alpha & \beta \\ \alpha & \beta \end{bmatrix} = \begin{bmatrix} \frac{\beta}{\alpha} & -1 \\ 1 & 1 \end{bmatrix} \begin{bmatrix} 1 & 0 \\ 0 & 1-(\alpha+\beta) \end{bmatrix} \begin{bmatrix} \frac{\beta}{\alpha} & -1 \\ 1 & 1 \end{bmatrix}^{-1}$$

Iterating the map yields:

$$\begin{bmatrix} f_1(k+1) \\ f_2(k+1) \end{bmatrix} = \begin{bmatrix} \frac{\beta}{\alpha} & -1 \\ 1 & 1 \end{bmatrix} \begin{bmatrix} 1 & 0 \\ 0 & (1-(\alpha+\beta))^{k} \end{bmatrix} \begin{bmatrix} \frac{\beta}{\alpha} & -1 \\ 1 & 1 \end{bmatrix}^{-1} \begin{bmatrix} f_1(0) \\ f_2(0) \end{bmatrix}$$

And since $f_1(j) + f_2(j) = 1$ for each j, in particular for $j = 0$:

$$\begin{bmatrix} f_1(k+1) \\ f_2(k+1) \end{bmatrix} = \begin{bmatrix} \frac{\beta}{\alpha+\beta} + \frac{f_1(0)\,\alpha - f_2(0)\,\beta}{\alpha+\beta} (1-(\alpha+\beta))^{k} \\ \frac{\alpha}{\alpha+\beta} + \frac{-f_1(0)\,\alpha + f_2(0)\,\beta}{\alpha+\beta} (1-(\alpha+\beta))^{k} \end{bmatrix}$$

Convergence is to the stationary distribution:

$$\begin{bmatrix} f_1 \\ f_2 \end{bmatrix} \to \begin{bmatrix} \frac{\beta}{\alpha+\beta} \\ \frac{\alpha}{\alpha+\beta} \end{bmatrix}$$

The rate of convergence is easily seen to be exponential and modulated by the parameters α and β. We can further find the half life associated to the effects of the second eigenvalue in terms of α and β by solving the following for k:
log(0.5) = k log(1-($\alpha + \beta$)).
In simulation these formulas are useful to determine a ***burn-in*** period for the simulation, burn-in is the time frame required for the populations to reach a nearly stationary distribution. The queuing process involving cellularization at rate α *and* decellularization at rate β enables the simulation to explore the essential effects of signaling game theory in in the various states of endo-symbiosis while maintaining a fixed and constant size population of the total number of sender/receiver agents. This approach further allows for simpler initialization where the initial population are random independent sender and receiver agents only, the endo-pairs eventually arise and reach stationarity after burn-in is completed.

In our simulation since α and β are 0.0001 and 0.01, the stationary distribution will be .9901 of encounter pairs will be in extracellular condition, while ~0.01 are in cellular condition. In terms of burn-in, given values of α and β as 0.0001 and 0.01, we can determine that at time *k~68* the population is at halflife where the effects of initial population are half and the distribution of proportion of types is giving way to its stationary distribution.
